# Influence of Transportation Noise and Noise Sensitivity on Annoyance: A Cross-Sectional Study in South Korea

**DOI:** 10.3390/ijerph14030322

**Published:** 2017-03-20

**Authors:** Joo Hyun Sung, Jiho Lee, Kyoung Sook Jeong, Soogab Lee, Changmyung Lee, Min-Woo Jo, Chang Sun Sim

**Affiliations:** 1 Department of Occupational and Environmental Medicine, Ulsan University Hospital, University of Ulsan College of Medicine, 877 Bangeojinsunhwando-ro, Dong-gu, Ulsan 44033, Korea; yadaf@hanmail.net (J.H.S.); oemdoc@naver.com (J.L.); 2 Department of Occupational and Environmental Medicine, Dongguk University Seoul, Graduate School of Medicine, 27 Dongguk-ro, Ilsandong-gu, Goyang-si, Gyeonggi-do 10326, Korea; bandyoem@naver.com; 3 Department of Mechanical and Aerospace Engineering, Seoul National University, 1 Gwanak-ro, Gwanak-gu, Seoul 08826, Korea; solee@snu.ac.kr; 4 School of Mechanical Engineering, University of Ulsan, 93, Daehak-ro, Nam-gu, Ulsan 44610, Korea; cmlee@ulsan.ac.kr; 5 Department of Preventive Medicine, University of Ulsan College of Medicine, 88, Olympic-ro 43-gil, Songpa-gu, Seoul 05505, Korea; mdjominwoo@gmail.com

**Keywords:** transportation noise, annoyance, sensitivity, health impact assessment

## Abstract

Environmental noise is known to cause noise annoyance. Since noise annoyance is a subjective indicator, other mediators—such as noise sensitivity—may influence its perception. However, few studies have thus far been conducted on noise annoyance in South Korea that consider noise sensitivity and noise level simultaneously. The aim of this study was to evaluate the correlations between noise sensitivity or noise level and noise annoyance on a large scale in South Korea. This study estimated the level of noise exposure based on a noise map created in 2014; identified and surveyed 1836 subjects using a questionnaire; and assessed the impact of transportation noise and noise sensitivity on noise annoyance. The result showed that noise exposure level and noise sensitivity simultaneously affect noise annoyance, and noise sensitivity has a relatively larger impact on noise annoyance. In conclusion, when study subjects were exposed to a similar level of noise, the level of noise annoyance differed depending on the noise sensitivity of the individual.

## 1. Introduction

Environmental noise is defined as “unwanted or harmful outdoor sound created by human activities” [1]. This includes transportation noise caused by airplanes, automobiles, or trains; neighbourhood noise; and leisure noise [2]. Environmental noise is known to cause a wide array of health problems. The World Health Organization (WHO) reported on such health problems as tinnitus, cardiovascular disease, child cognitive disabilities, sleep disorder, and noise annoyance in 2011 [3].

Among those health problems, noise annoyance is defined as “a feeling of displeasure caused by noise” [4]. As noise annoyance has a recommended threshold and shows a dose-response relationship, it has been widely used in assessing the health effects of environmental noise [5,6]. Since noise annoyance is a subjective indicator, it is affected not only by the level of noise exposure, but also by other mediators, including fear of danger from the noise source [7,8], noise preventability [7], attitude towards the noisy situation [9], and noise sensitivity [7,8,9,10]. Among these mediators, noise sensitivity is defined as “a factor involving underlying attitudes towards noise in general” and is also known to affect noise annoyance; many studies have suggested considering noise sensitivity together when analysing noise annoyance [2,11,12,13,14,15,16]. Few studies have thus far been conducted on noise annoyance in South Korea that consider noise sensitivity and noise level simultaneously, and there have been few investigating a large-scale population.

This study aims to examine correlations between noise sensitivity or noise level and noise annoyance by using data about transportation noise exposure from a community-dwelling setting of a local population on a large scale.

## 2. Materials and Methods

### 2.1. Study Population

**Figure 1:**
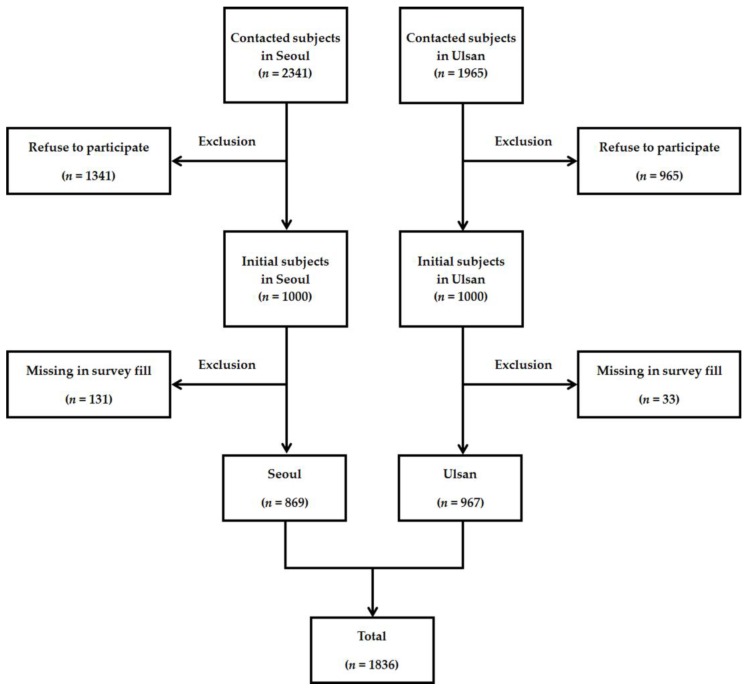
Flowchart of subject selection criteria.

The study site selected was Yangcheon-gu in Seoul and Nam-gu in Ulsan, areas for which we completed noise maps in 2014. Based on the noise maps, we stratified the buildings in those selected districts into four levels based on noise level (below 50 dBA, 50–59.9 dBA, 60–69.9 dBA, and above 70 dBA), then grouped them into similar areas. We determined the sample size for each level based on the size of the population. In order to extract households at the same probability, the study used a local sampling method and recruited 1000 subjects in Seoul and Ulsan, respectively. Until the required sample size was achieved, we contacted 2341 subjects in Seoul and 1965 subjects in Ulsan by using available methods (mainly home visiting, e-mail, and phone calls). When we contacted subjects and accounted for this study, if subjects refused to participate then we excluded those subjects. We visited subjects’ houses, described the object of this study, provided guidance, and obtained written consent. We conducted face-to-face interviews using computer-assisted personal interviewing (CAPI) to reduce the missing rate. We administered the questionnaire survey from July 2015 to January 2016. Out of 2000 possible subjects, 1836 were included in the study after excluding 164 whose questionnaire answers were missing (Figure 1).

### 2.2. Survey

The questionnaire included questions on social and demographic information as well as questions on noise sensitivity and noise annoyance. Social and demographic variables included age, sex, education level, marital status, monthly income, smoking status, alcohol drinking, exercise, and length of time at the present residence. Education level was divided into high school graduation or lower and two-year college graduation or higher; marital status was divided into married and single; and the monthly income was divided into less than 3 million KRW and at least 3 million KRW. Smoking status was divided into current smoker and current non-smoker (including past smoker and non-smoker); current smokers were defined as those who smoked currently; past smokers were defined as those who smoked more than 100 cigarettes over their lifetime and did not smoke currently; non-smokers were defined as those who smoked less than 100 cigarettes over their lifetime and did not smoke currently [17]. Alcohol drinking was categorized into current drinker and current non-drinker, and exercise status was categorized into regular exerciser and non-regular exerciser.

To assess noise sensitivity and noise annoyance, we used an 11-point visual analog scale (VAS) ranging from 0 to 10 that we created based on the International Organization for Standardization Technical Specification (ISO/TS) 15666 (2003) [18]. For noise sensitivity, subjects exceeding the average scale value of the total subjects were classified as “high sensitivity (6–10 points)” group, and others classified as the “low sensitivity (0–5 points)” group. For noise annoyance, subjects exceeding 72% of the point scale (8–10 points) were classified as the “highly annoyed” group, while subjects exceeding 50% of the point scale (6–10 points) were classified as the “annoyed” group [6].

### 2.3. Transportation Noise Levels

In order to estimate the noise level of the residential districts in which the subjects resided, this study used a noise map that we created in 2014. With data from the noise map, we used noise prediction software (Cadna A, DataKustik, Gilching, Germany) to calculate the transportation noise levels at the exterior wall of the residential buildings of the study subjects, based on addresses confirmed during the questionnaire survey. The number of passing vehicles per hour and the percentage of heavy vehicles per hour are the main input variables for the calculation of road traffic noise. Those values are measured for each time interval on the real road. Additionally, road shape, road surface, barriers by the roads, and the speed limit of the road are included for the input values. Furthermore, the geographical and meteorological inputs are used, such as three-dimensional building polygons, contour lines, and annual temperature and pressure values. The verification of the noise map was conducted by measuring twenty points of the study area and comparing the calculated values to the measured values. If the difference between calculated values and measured values was less than 3 dB, then the noise map was considered reliable for use in the study.

This study used the day–night average sound level (L_dn_) as a noise indicator. The low-noise group and the high-noise group were divided based on the threshold at which environmental noise could pose a risk to health [19]; 55 dBA—the average transportation noise in the roads of the study districts—was set as the threshold in this study. Subjects were classified as the low noise group when their noise exposure level was less than 55 dBA, while subjects were classified as the high noise group when their level of exposure was 55 dBA or higher.

### 2.4. Statistical Analysis

We performed a Pearson’s correlation analysis to look into correlations between noise level or noise sensitivity and noise annoyance. We performed multiple linear regression analysis in order to check multi-collinearity between noise level and noise sensitivity. To compare the ratio of those “highly annoyed” and “annoyed” based on noise level and noise sensitivity, we conducted a chi-square test. Based on noise level and noise sensitivity, we categorized the subjects into four combinations: “low sensitivity/low noise”, “low sensitivity/high noise”, “high sensitivity/low noise”, and “high sensitivity/high noise”. To compare age and length of time at the present residence according to the four combinations, this study used analysis of variance (ANOVA) and Tukey’s method for post-hoc verification. To compare sex, education level, marital status, monthly income, smoking status, alcohol drinking, exercise, highly annoyed, and annoyed, we performed a chi-square test.

We conducted logistic regression to show an interaction by modelling interaction variables (noise sensitivity × noise exposure). We also conducted multiple logistic regressions to calculate the adjusted odds ratio (aOR) to adjust for confounders that could affect annoyance (age, residential period, sex, education level, marital status, monthly income, smoking status, alcohol drinking, and exercise).

We used SPSS 21.0 (IBM SPSS Inc., Chicago, IL, USA) to analyse all data. The significance level was set at 0.05, and we considered a *p*-value of less than 0.05 to be significant.

### 2.5. Ethics

This study was approved by the Institutional Review Board of Ulsan University Hospital (IRB No. 2014-08-008). This study has been conducted from 2014 to the present. From 2015, we examined the health effects of environmental noise on humans. All participants took part in this study voluntarily and written consent was obtained from participants.

## 3. Results

The results of the correlation analysis between noise sensitivity, noise level, and noise annoyance to transportation noise showed that the correlation coefficient between noise sensitivity and noise annoyance was 0.39 (*p* < 0.001) while the correlation coefficient between noise level and noise annoyance was 0.20 (*p* < 0.001), each of which showed a positive correlation. There was no multi-collinearity between noise level and noise sensitivity in the results of multiple linear regression. We have not presented the results of regression as a table.

The general characteristics of all subjects and the four combination groups are presented in Table 1. The average age of subjects was 47.0 ± 16.1 years; the average residence period was 9.1 ± 8.5 years; and the average noise exposure level was 55.2 ± 10.4 dBA. After the characteristics of the four combination groups were analysed, we found that the average age was higher in the two high noise sensitivity groups than the two low noise sensitivity groups (*p* = 0.019). The average residence period was also longer in the two high sensitivity groups than the two low sensitivity groups (*p* = 0.009). The proportion of women was higher in the two high sensitivity groups than the two low sensitivity groups (*p* < 0.001), while the education level was lower in the two high sensitivity groups than the two low sensitivity groups (*p* = 0.001). The monthly income was higher in the two high noise groups than the two low noise groups (*p* < 0.001). The proportion of both smoking status and regular exercise was higher in the two low noise groups than the two high noise groups (*p* < 0.001, Table 1).

The proportion of “highly annoyed” and “annoyed” by noise exposure showed an increasing trend as noise exposure increased. This trend also appeared in noise sensitivity, but the proportion was higher in the noise sensitivity group than the noise exposure group. When the four combinations of “low sensitivity/low noise”, “low sensitivity/high noise”, “high sensitivity/low noise”, and “high sensitivity/high noise” were categorized in consideration of noise exposure level and noise sensitivity together, and the proportion of “highly annoyed” and “annoyed” for those four groups were analysed, it was found that the proportion of the “highly annoyed” for each group was 4.2%, 6.6%, 15.3%, and 23.0% (*p* < 0.001), respectively, while the proportion of “annoyed” was 13.8%, 22.0%, 41.7%, and 55.2% (*p* < 0.001, Table 2), respectively.

**Figure 2:**
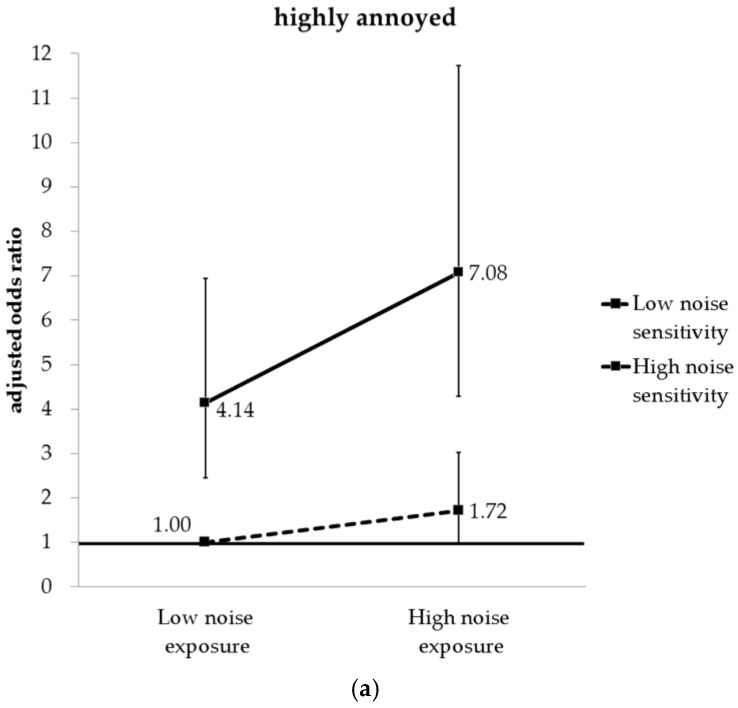

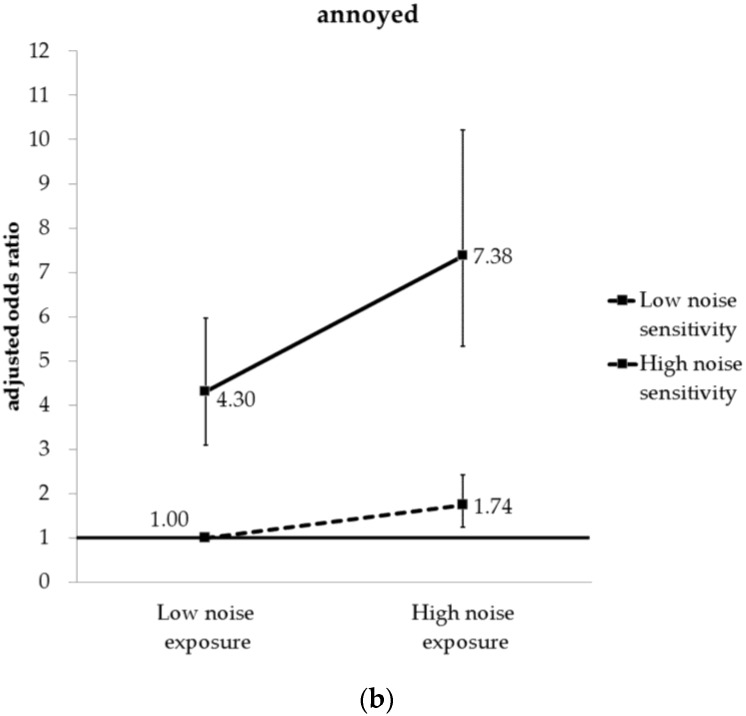
Adjusted odds ratio of being (**a**) “highly annoyed” and (**b**) “annoyed” according to noise sensitivity and noise exposure.

A model that considered the interaction variables (noise sensitivity × noise exposure) showed statistical significance in “highly annoyed” (OR 3.37; 95% CI 2.51–4.53) and “annoyed” (OR 3.81; 95% CI 3.03–4.78) groups. After analysing the risk of annoyance in consideration of both noise level and noise sensitivity, the aOR of being “highly annoyed” in “low sensitivity/high noise”, “high sensitivity/low noise”, and “high sensitivity/high noise” was 1.72 (95% CI 0.98–3.02), 4.14 (95% CI 2.46–6.99), and 7.08 (95% CI 4.28–11.73), respectively, compared to the “low sensitivity/low noise” group. The aOR of being “annoyed” for those groups was 1.74 (95% CI 1.25–2.42), 4.30 (95% CI 3.10–5.97), and 7.38 (95% CI 5.33–10.21), respectively (Figure 2).

## 4. Discussion

To assess the noise annoyance of transportation noise, this study analysed data from 1836 residents in Yangcheon-gu, Seoul, and Nam-gu, Ulsan which were located on a developed noise map, and compared noise annoyance depending on the noise level. The average noise level estimates based on residential districts on the noise map were 55.2 ± 10.4 dBA (ranging from 46.0 ± 5.7 to 64.0 ± 5.7 dBA). This noise level was not as high as occupational noise that reaches about 90 dBA [2], so noise sensitivity would have a greater impact on noise annoyance [10]. In this respect, this study stratified the subjects according to noise level, noise sensitivity, and noise level and noise sensitivity together, and analysed their impact on noise annoyance, respectively.

Initially, we performed a correlation analysis to verify correlations between noise level or noise sensitivity and noise annoyance. The results showed that there were significant correlations between the two variables and noise annoyance, and that the correlation coefficient between noise sensitivity and noise annoyance (0.39) was higher than that between noise level and noise annoyance (0.20). When the proportion of “highly annoyed” and “annoyed”—depending on noise level or noise sensitivity—was analysed, the higher noise level group and the higher noise sensitivity group showed a higher proportion of “highly annoyed” and “annoyed”, but the difference was much larger for noise sensitivity. Past studies reported that differences in noise annoyance depending on noise level were not distinctive when there was a low level of noise exposure, but noise sensitivity had a larger impact on noise annoyance when there was a low level of noise exposure [10,20,21]. The reason behind such results could be that noise annoyance—the indicator we used in this study—is a subjective indicator, and it could be affected by noise sensitivity—a subjective characteristic [13,16,22].

Based on our initial findings, we re-classified the subjects into four groups in consideration of the noise level and the noise sensitivity together, and analysed the proportion of “highly annoyed” and “annoyed”. The results showed that the proportion of “highly annoyed” and “annoyed” increased in the order of “low sensitivity/low noise”, “low sensitivity/high noise”, “high sensitivity/low noise”, and “high sensitivity/high noise”. Furthermore, the results of multiple logistic regression analysis showed that the aOR of being “highly annoyed” and “annoyed” tended to gradually increase in the order of “low sensitivity/high noise”, “high sensitivity/low noise”, and “high sensitivity/high noise” compared to the “low sensitivity/low noise” group. Although many previous studies found that noise level and noise sensitivity affected noise annoyance, most of those studies presented results regarding analysis of correlations only [2,10,22]. Unlike the methods of the past studies, this study stratified subjects according to noise level and noise sensitivity, re-classified subjects into four groups, and analysed the impact on noise annoyance. We found that noise level and noise sensitivity simultaneously affect noise annoyance, and when we analysed with four groups, the impact of noise sensitivity on noise annoyance was more prominent than that of the noise level. In addition, although exposed to a similar level of transportation noise on the road, reactions to noise annoyance differs depending on noise sensitivity. Therefore, if noise level were considered alone when assessing the impact of transportation noise on noise annoyance, there could be a possibility of underestimating the impact of noise.

Moreover, in the results of the general subject characteristics, it was found that higher noise sensitivity was correlated with relatively higher age, lower education level, and female sex. Even though there have been few studies looking into factors that impact noise sensitivity, we could find that a previous study found similar results [23,24]. In summary, noise sensitivity was higher among those who could be considered a relatively vulnerable group, and noise annoyance was higher, although they were exposed to a similar level of transportation noise as those who reported low noise annoyance.

Thus, we found that noise level and noise sensitivity simultaneously affect annoyance, and noise sensitivity has a relatively larger impact on noise. Furthermore, as seen in this study’s results, when noise sensitivity was considered together with noise level, the impact on annoyance could be assessed in more detail for similar levels of noise exposure. In addition, unlike the industrial workplace population comprising mostly physically healthy workers, environmental noise—including transportation noise—could impact a vulnerable group who could have relatively higher noise sensitivity [23,24,25]. Therefore, if noise sensitivity is considered with noise together when assessing not just noise annoyance, but also other health impacts of environmental noise, it would ensure a more appropriate health assessment.

This study has some limitations. First, it was a cross-sectional study that could only evaluate correlations between noise level or noise sensitivity and noise annoyance, and was not able to verify causal relationships or assess long-term exposure. Second, we used an 11-point VAS scale based on ISO/TS 15666 (2003) to assess noise sensitivity because there is no universally used simple noise sensitivity scale. Thus, an absolute cut-off value would be inaccurate, nevertheless we used the average value of the subjects as a cut-off value because we thought it is reasonable. Third, assessment of noise annoyance—one of the most widely used indicators to evaluate the health impact arising from environmental noise exposure—is generally conducted based on a questionnaire (a subjective indictor), and an objective assessment method to support the questionnaire has thus far not been established. Therefore, self-report bias could have occurred on the questionnaire survey in this study. Fourth, this study was funded by the Korean Ministry of Environment (MOE), and the MOE did not want to cover extreme noise levels. Thus, the noise level ranged from 46 to 64 dBA, and we could not assess annoyance at higher noise levels.

Nevertheless, this study has several important implications. First, it is the first study in South Korea that has assessed the health impact of environmental noise on noise annoyance in a large-scale study of a population exposed to transportation noise in their daily lives. Second, while previous studies mostly focused on assessing noise annoyance depending on noise level, this study considered noise sensitivity as well. Thus far, there have been few studies on the health impact of environmental noise in South Korea that assessed noise sensitivity and environmental noise levels together. Therefore, this study could be meaningful in that it is the first large-scale study in South Korea that considers noise level and noise sensitivity in assessing noise annoyance.

## 5. Conclusions

In conclusion, we could see that when a population is exposed to a similar level of noise, the level of noise annoyance varies depending on noise sensitivity—especially with relatively low noise levels, such as environmental noise. When other variables that could affect the subjective assessment are controlled, the results are identical. Therefore, a future study on the health impact of environmental noise needs to consider not only the physical effects of noise, but also individuals’ noise sensitivity.

## Figures and Tables

**Table 1:** General subject characteristics.

Variables	Categories	Total (*n* = 1836)	Low Noise Sensitivity		High Noise Sensitivity		*p*-Value
			Low Noise Exposure ^a^ (*n* = 501)	High Noise Exposure ^b^ (*n* = 527)	Low Noise Exposure ^c^ (*n* = 386)	High Noise Exposure ^d^ (*n* = 422)	
Age (years) *		47.0 ± 16.1	46.0 ± 16.7	46.0 ± 16.0	48.5 ± 16.6	48.2 ± 14.9	0.019
Residence period (years) ^†^		9.1 ± 8.5	8.6 ± 8.6	8.6 ± 7.5	10.3 ± 9.6	9.2 ± 8.3	0.009
Noise level (dBA) **		55.2 ± 10.4	46.0 ± 5.7	63.4 ± 5.3	46.5 ± 5.7	64.0 ± 5.7	<0.001
Sex	Men	696 (37.9)	222 (44.3)	211 (40.0)	126 (32.6)	137 (32.5)	<0.001
	Women	1140 (62.1)	279 (55.7)	316 (60.0)	260 (67.4)	285 (67.5)	
Education level	High school and less	858 (46.7)	219 (43.7)	227 (43.1)	213 (55.2)	199 (47.2)	0.001
	College and more	978 (53.3)	282 (56.3)	300 (56.9)	173 (44.8)	223 (52.8)	
Marital status	Single	733 (39.9)	237 (47.3)	217 (41.2)	155 (40.2)	124 (29.4)	<0.001
	Married	1103 (60.1)	264 (52.7)	310 (58.8)	231 (59.8)	298 (70.6)	
Monthly income	<3000	729 (39.7)	248 (49.5)	179 (34.0)	172 (44.6)	130 (30.8)	<0.001
(1000 KRW)	≥3000	1107 (60.3)	253 (50.5)	348 (66.0)	214 (55.4)	292 (69.2)	
Smoking status	Non-smoker	1591 (86.7)	400 (79.8)	470 (89.2)	334 (86.5)	387 (91.7)	<0.001
	Smoker	245 (13.3)	101 (20.2)	57 (10.8)	52 (13.5)	35 (8.3)	
Alcohol drinking	No drink	934 (50.9)	237 (47.3)	266 (50.5)	213 (55.2)	218 ( 51.7)	0.135
	Drink	902 (49.1)	264 (52.7)	261 (49.5)	173 (44.8)	204 (48.3)	
Regular exercise	No	691 (37.6)	155 (30.9)	211 (40.0)	127 (32.9)	198 (46.9)	<0.001
	Yes	1145 (62.4)	346 (69.1)	316 (60.0)	259 (67.1)	224 (53.1)	

Unit: mean ± standard deviation, number (percentage); ^a^ Low noise sensitivity and low noise exposure; ^b^ Low noise sensitivity and high noise exposure; ^c^ High noise sensitivity and low noise exposure; ^d^ High noise sensitivity and high noise exposure; * post hoc comparison using Tukey’s method: a,b < c,d; ^†^ post hoc comparison using Tukey’s method: a,b < c; ** post hoc comparison using Tukey’s method: a,c < b,d.

**Table 2:** Proportion of highly annoyed and annoyed according to noise exposure, noise sensitivity, and a complex of noise sensitivity and exposure.

Variables	Highly Annoyed	Annoyed
Low NS ^a^ (*n* = 1028)	56 (5.4) *	185 (18.0) *
High NS (*n* = 808)	156 (19.3)	394 (48.8)
Low NE ^b^ (*n* = 887)	80 (9.0) ^†^	230 (25.9) *
High NE (*n* = 949)	132 (13.9)	349 (36.8)
Low NS + low NE (*n* = 501)	21 (4.2) *	69 (13.8) *
Low NS + high NE (*n* = 527)	35 (6.6)	116 (22.0)
High NS + low NE (*n* = 386)	59 (15.3)	161 (41.7)
High NS + high NE (*n* = 422)	97 (23.0)	233 (55.2)

Unit: number (percentage); ^a^ NS, noise sensitivity; ^b^ NE, noise exposure; * *p* < 0.001; ^†^ *p* = 0.001.
